# Teaching epistemic integrity to promote reliable scientific communication

**DOI:** 10.3389/fpsyg.2024.1308304

**Published:** 2024-04-05

**Authors:** Aurélien Allard, Christine Clavien

**Affiliations:** Faculty of Medicine, Institute for Ethics, History, and the Humanities, University of Geneva, Geneva, Switzerland

**Keywords:** research integrity education, epistemic virtue, moral education, misinformation, epistemic skills

## Abstract

In an age of mass communication, citizens need to learn how to detect and transmit reliable scientific information. This need is exacerbated by the transmission of news through social media, where any individual has the potential to reach thousands of other users. In this article, we argue that fighting the uncontrolled transmission of unreliable information requires improved training in broad epistemic integrity. This subcategory of research integrity is relevant to students in all disciplines, and is often overlooked in integrity courses, in contrast to topics such as fraud, plagiarism, collaboration and respect for study subjects. Teaching epistemic integrity involves training epistemic *skills* (such as metacognitive competences, capacity to use helpful heuristics, basic statistical and methodological principles) and *values* (such as love of truth, intellectual humility, epistemic responsibility). We argue that this topic should be addressed in secondary school, and later constitute a fundamental component of any university curriculum.

## 1 Introduction

The last few years have seen increased attention paid to fake news, or to the widespread transmission of scientific misinformation through social media and politicized media channels. Journalists and researchers have evoked the rise of “infodemics” (Gallotti et al., 2020). These fears are probably exaggerated and there are ongoing debates about the extent to which misinformation is a problem (Guess et al., 2019; Adams et al., 2023). Nevertheless, they highlight the need for improved scientific education for all in order to reduce deliberately promoted or accidentally shared misinformation (Southwell et al., 2018). In an age of mass communication, all citizens are involved to some extent in the production and sharing of information. When people share news on Twitter, WhatsApp, or any social media, their actions can have far-reaching consequences in terms of the propagation of accurate or inaccurate news (Naeem et al., 2021).

In this paper, we argue that teaching epistemic integrity constitute a basis for improving our collective ability to evaluate and share scientific information. As future citizens, students need to learn how to recognize reliable information, and to become motivated to transmit it in the most accurate and informative way to other people. These competences involve the development of skills and virtues that are as crucial in daily life as they are for science.

While the need for improved epistemic skills is made most salient by the accelerated transmission of information via social media, the benefits that would accrue from such training are much broader. All citizens evaluate information and reasons on a daily basis, whether they are online or offline. For instance, they receive health-related news, evaluate and transmit them to friends or family, and sometimes base their political decisions on that information (Vosoughi et al., 2018). Teaching citizens how to recognize reliable information, and how to be careful in transmitting it, are fundamental processes that remain crucial beyond the phenomenon of social media (Van Damme et al., 2022).

Recent research on the treatment of misinformation has stressed the need to improve digital or statistical literacy of students and citizens (Guess et al., 2020; Howell and Brossard, 2021). This is a necessary step, but we worry that it may be insufficient if not complemented by training *further epistemic skills* (e.g., metacognitive skills, awareness of cognitive biases, basic knowledge of scientific methods, or recognition of signs of scientific reliability), and *epistemic virtues* (e.g., intellectual humility and honesty, love of truth, or epistemic responsibility). This concern echoes recent work in applied virtue epistemology, where philosophers have criticized the traditional focus of critical thinking courses on teaching *skills*, such as logic and argumentation, at the expense of teaching epistemic *virtues*, such as open-mindedness or love of truth (Battaly, 2006; Baehr, 2013, 2017). People need to think and act like virtuous scholars when treating complex information. This is why training basic epistemic *skills* and complementary epistemic *virtues* should be part of dedicated classes. We call this double focus “broad epistemic integrity.”

## 2 Epistemic integrity: a crucial component of responsible conduct of research

Research integrity is mostly taught in disciplines specialized in human or animal research (medicine, psychology, or biology). It constitutes a very broad domain, and includes such diverse issues as plagiarism, fraud, collaboration in a research context, or the ethical treatment of research participants. Following philosopher Heather Schroeder-Heister et al. (2015), the requirements of responsible conduct of research can be divided in three broad categories: (1) norms regulating the research ecosystem (i.e., norms regulating cooperation: authorship, plagiarism, etc.), (2) moral norms in the treatment of research participants (protecting subjects), and (3). norms promoting the proper function of science, i.e., the production of reliable knowledge.

The third set of norms regulates the collection of existing information (e.g., literature search), the collection of raw data about the world, the testing of hypotheses about the world, the interpretation of information, the evaluation of research results, and the transmission of this information to outside observers (Schroeder-Heister et al., 2015). The aim is to secure rigour in data collection, impartiality in analysis, and promote an unbiased transmission of information, without distorting it by either hyping it or bending it toward some particular ideological stance.

Many aspects of this third category of norms (promoting the proper function of science) also serve to regulate the transmission of reliable knowledge in daily life. This indicates that some requirements of responsible conduct of research are relevant to a broader population than science students. Even though most citizens do not conduct original research, they must be able to dig through a confusing array of news and must be able to transmit reliable information. Activities central to the scientific process such as the evaluation and communication of information are thus played out in ordinary life in modern democracies. Since such activities have a societal impact, citizens have a duty to transmit reliable science. Since this is not easy to achieve, schools and universities have a duty to teach the necessary skills and virtues for “broad epistemic integrity”: student-citizens should realize that they have a duty to be honest communicators, and that this requires learning how to recognize reliable knowledge, which in turn requires understanding how reliable knowledge is created.

## 3 The cognitive and moral dimensions of broad epistemic integrity

As illustrated in Table 1, broad epistemic integrity involves two types of abilities: *epistemic skills* that facilitate the detection of reliable sources, and *epistemic virtues*, such as love of truth and epistemic responsibility. This partition echoes pioneering work of authors who argue that cognitive skills and affective dispositions are necessary dimensions of critical thinking (e.g., Facione, 2000; Andreucci-Annunziata et al., 2023). In what follows, we describe what could be included in such a broad epistemic integrity course.

**Table 1:** Summary of important content for teaching broad epistemic integrity.

Epistemic *skills*	Metacognitive competences
	Awareness of cognitive biases
	Critical use of heuristics to identify reliable sources
	Basic understanding of scientific methods (importance of randomization, blind testing, rigour in sampling and data collection, etc.) and statistics (importance of sample size, regression to the mean, etc.)
	Understanding of the logical structure of arguments and vigilance regarding the link between evidence and conclusions
Epistemic *virtues*	Love of truth
	Intellectual humility
	Open-mindedness and search for impartiality
	Epistemic responsibility

### 3.1 Epistemic skills

Non-specialists often have a hard time navigating the complexity of the scientific literature. It is nevertheless possible to improve students’ abilities in this regard. The epistemic skills students need to learn include at least fundamental metacognitive competences, awareness of cognitive biases, and the capacity to assess both the reliability of the *source* and the *content* of the information. Let us study each of these components in turn.

First, background metacognitive skills and knowledge need to be acquired (Kuhn, 1999). It involves the capacity to make basic epistemological distinctions between *beliefs* which are generated by human minds (own assertions or claims made by others), and the *evidence* in favour or contra those beliefs. Evidence may be provided by direct observation of the world, or by some form of investigations (correlational or theory-based observations). Further, students need to keep track of how they have obtained information, and to evaluate the epistemological status of the different sources of their beliefs. For instance, obtaining information reported by an unknown person does not bear the same epistemic value as direct observation. Moreover, students may be taught that the process of learning is plagued by numerous of cognitive biases, such as social conformity bias (Whiten, 2019), preference for positive results (Allard and Clavien, 2023), or belief perseverance when a coherent causal story supports the belief (Green and Donahue, 2011). To sum up, students need to understand that not all opinions are equal, and that knowing is a process that entails judgment, evaluation, arguments, and critical scrutiny.

Based on these background metacognitive skills, students can be taught how to recognize whether a *source* is reliable. This is a complex task for outsiders to the academic field. Since most students lack the background knowledge learned during participation in the research activity itself and shared at lab meetings or other scientific events, they need to learn practical ways to crosscheck assertions and to conduct web-based inquiries on the reputation of actors (Wineburg and McGrew, 2017). First they can learn how to track conflicts of interest and how to evaluate when those conflicts are likely to bias the scientific quality and the results (Gorman, 2018). Second, they can learn to reconstruct the epistemic standing of actors based on a few rough heuristics (Origgi, 2017; Kim et al., 2019). Traditional signs of prestige, such as journal impact factor, an article citation count, or university rankings of authors have been widely criticized (Brembs et al., 2013; Brembs, 2018). They are not perfect predictors of quality. However, for someone who lacks specific competencies in the scientific field and who needs to make an evaluation in constrained circumstances (little time, no specialists available to answer questions), these signs of prestige can nevertheless be used as useful heuristics (Bordons et al., 2002). Students need, however, to keep a critical view on these heuristics. For this, they need to get a rough idea of the structure of a scientific field and of how scientific evidence is produced, challenged, revised and refined in a step procedure characterized by organized scepticism (Merton, 1979). This will help them to understand why, in science, impact factors and rankings are indications of scientific reliability (they reflect systematic quality screening of peer review processes), although they should be used carefully. Notably, these signs of prestige are often unreliable in peripheral countries (whose researchers do not exclusively publish in English or whose national journals are insufficiently covered by ISI databases), or for making inter-field comparisons, and they do not immunize against wrong and sometimes fraudulent results, which may become pervasive and difficult to correct when they emanate from prestigious institutions (Bordons et al., 2002).

These heuristics are too rough to be blindly trusted, however. We also need to teach students how to evaluate the reliability of the *content* of articles. This implies learning the basics of scientific methods. For instance, some statistical knowledge needs to be acquired, such as the value of high sample sizes or the use of proper randomization. In a non-mathematical way, students should develop intuitions about common threats to statistical conclusions, such as the risk of confounding, selection bias, or the difference between correlation and causation. Psychological research has consistently shown that statistical training can improve judgments about everyday situations, and can even improve accuracy in predicting world events (Fong et al., 1986; Nisbett et al., 1987; Mellers et al., 2014; Chang et al., 2016). Learning basic statistical skills should thus enable students to recognize not only *fake news* but also *misleading news*, as for instance reports of surveys or experiments that do not properly control for confounds. In an ideal world, real news about dubious research should be evaluated severely by citizens. Basic aspects of scientific methodology are crucial for any student, even those who will not use such methods themselves (e.g., students in the humanities). In a context of growing interdisciplinarity (Buyalskaya et al., 2021), being able to recognize reliable theories in other fields should be considered priority.

Beyond strictly statistical topics, students should learn to carefully evaluate the evidential basis for any claim proposed in a study. This implies being able to recognize the articulation of arguments, and to evaluate whether a conclusion follows from its premises. If a new study makes counter-intuitive or bold claims, students must be able to systematically evaluate whether conclusions follow from the evidence offered (El Soufi and See, 2019).

### 3.2 Epistemic virtues

Teaching epistemic skills is not enough, because humans are prone to biases that can distort their treatment of information even in contexts where citizens possess *in theory* the necessary skills to process information. First, there is a substantial disconnect between what people believe and what they share on social media. Research on the psychology of fake news transmission has shown a gap between the ability to distinguish fake and real news and the tendency to share fake news (Bor et al., 2020; Sirlin et al., 2021). People sometimes share fake news even when they do not believe it. Second, and most importantly, students are likely to be influenced by political, religious or cultural biases. It is therefore crucial that our teaching also includes training in specific moral attitudes and values related to the objective assessment of information. Research integrity involves teaching students a specific mindset: learning to evaluate information not in terms of the promotion of specific goals (will it bolster my worldview?) but in terms of the trustworthiness of the information.

The *love of truth* for its own sake might seem an obvious virtue, and we might expect students to already possess it. In one sense or another, everybody values truth. But what we need to teach is that truth should be valued even when it goes against other dearly held commitments. Students need to learn how to overcome their own motivated reasoning and accept true propositions even when they contradict their political, religious or cultural beliefs. Without this love of truth, regardless of personal convictions and identity, it is difficult to address polarized debates in academic and civic contexts (Kahan et al., 2012).

A complementary epistemic virtue is *intellectual humility* (Marie and Petersen, 2022). We have in previous sections highlighted the importance of heuristics. However, heuristics can be dangerous. Followed blindly, they can lead students to become overconfident in their judgment. For instance, while hearing about the importance of randomization, students should not learn to equate good methodology with the use of Randomized Controlled Trials. There can be good reasons for using a different method (Smith and Pell, 2003). Conversely, randomized studies can be of poor quality if they do not fulfil other criteria of scientific validity (Shadish et al., 2001). This is one more reason to teach students that heuristics are useful but not fully reliable, and that there is no perfectly secure way to ensure that a specific claim is true. Beyond learning the drawbacks of simple heuristics, humility would help students to learn the limits of their own worldview. Indeed, recent psychological research has shown that people high in epistemic humility have a lower tendency to share fake news hostile to outgroup members (Marie and Petersen, 2022), and overconfidence has been linked to a higher tendency to share fake news (Lyons et al., 2021). Students need to learn the intellectual humility necessary to acknowledge that, even though science can produce better-than-average claims, these claims will never be fully secure. But wielded with humility, heuristics for identifying reliable science can greatly help citizens and scientists to improve their judgments on complex debates, and to reinforce the complementary virtue of intellectual honesty.

Recent research in psychology has shown that actively open-minded thinking, the tendency to evaluate arguments based on their evidence, was one of the best predictors of the accurate evaluation of information (Scherer and Pennycook, 2020; Newton et al., 2023). A possible strategy (yet to be investigated) for fostering a commitment to *open-minded thinking and search for impartiality* could be to show students the risks of adopting a systematic political worldview. Students probably do not realize that their political or cultural values may influence their valuation of truth. However, it should be possible to show them that such conflicts are widespread by debunking widespread myths in a variety of domains. For instance, instructors could show the gap between expert opinion and widespread beliefs on different sides of the political spectrum. For instance, the lack of negative impact of immigration on the economy or unemployment could be contrasted with opposite common right-wing opinions (Banerjee and Duflo, 2019). On the other hand, instructors could also debate topics where common left-wing opinions are not aligned with expert opinion, such as the safety of nuclear power or the heritability of intelligence (Pew Research Center, 2015; Pennycook et al., 2023). If implemented in a fair and unbiased manner, such systematic debunking should lead students to realize the need to separate their cognitive beliefs from their political convictions.

In the context of the transmission of news on social media, an attachment to truth implies the virtue of *epistemic responsibility*. Epistemic responsibility involves transmitting information only if it seems plausible or reliable, and avoiding the promotion of unreliable research. People sometimes share fake news because the news flatters their own political worldview (Altay et al., 2021), even if they do not necessarily find the news particularly plausible (Sirlin et al., 2021). To be responsible agents, students need to understand the impact that their actions can have on other people’s beliefs. This implies teaching students that they are epistemic agents, and not simply epistemic recipients. Even when they are simply in a position of transmitting information (without producing it) they still can evaluate and select the information they transmit. In a teaching context, we need to make students realize that it is their duty to transmit accurate information, and that this is only possible if they pause before sharing news, and evaluate the accuracy of the source (Fazio, 2020).

## 4 Broad epistemic integrity in practice

Because it addresses skills and virtues highly relevant to all future citizens, the teaching of broad epistemic integrity should be available (or mandatory) to all students, including in disciplines such as biology, engineering, medicine, law, economics, humanities, political and social sciences, informatics, etc. Our proposition does not come in a vacuum: in some countries, there is a long history of classes teaching critical thinking to undergraduate students, and the recent focus on fake news has led to the development of additional digital literacy classes. For instance, many US universities propose a core academic curriculum that includes critical thinking classes. Courses closely related to our proposal are already proposed in some universities. Examples include the recent Calling Bullshit class taught at the University of Washington by Carl Bergstrom and Jevin West (Bergstrom and West, 2021), and fact-checking classes promoted in journalism schools. However, despite these examples, in most universities and high school curricula around the world (including high income European countries like France or Switzerland), the teaching of broad epistemic integrity is not integrated in an explicitly structured way, nor available to all students.

Depending on existing educational context, our proposition could stimulate the creation of a new course, or complementary units to existing initiatives or teaching. In theory, broad epistemic integrity could be integrated in methodological courses, such as scientific design, literature search, research integrity, critical thinking. However, to ensure sufficient content quality and time spent on the topic, we recommend developing it as a standalone course with dedicated teachers.

Moreover, to highlight its relevance from students’ point of view, teaching of broad epistemic integrity should be meaningfully embedded in each disciplinary curriculum. For instance, in scientific disciplines, it could be linked to classes on scientific methods, scientific literacy or research integrity. Or within a humanities curriculum, broad epistemic integrity would chime well with classes devoted to argumentation, logic, or analysis of discourse. Overall, our proposition should be seen as a complement rather than as a replacement of current teaching.

Proponents of virtue epistemology have proposed that students should learn epistemic virtues by modelling their attitudes on exemplars, i.e., individuals who are proper examples of epistemic virtues (Battaly, 2006; Baehr, 2013). In the context of a broad epistemic integrity class, two important categories of exemplars can be involved: the teacher of the class (ideally a trained tutor representative of students’ discipline, capable of addressing a wide range of controversial topics, and of showing open-mindedness and attachment to truth), and invited scientists or other scholars (sociology, political science) that can provide exemplary experience on epistemic integrity issues with broad societal impact. While discussing real cases, students can take up the role of a scientist searching to identify sound sources of information, and thereby, train their epistemic skills and virtues in group works, joint learning, and interactive feedback processes (for more insights on the methods that could be used to reach the pedagogical goals, see Andreucci-Annunziata et al., 2023).

Moreover, to increase transferability in the real world, and to bridge the gap between the academic world and the everyday transmission of information, we consider it crucial that students are trained to evaluate everyday content, including scientific news transmitted in social media and popular information vectors (newspaper articles, videos, etc.). Students should assess a wide range of information in a wide range of contexts, ranging from articles published in academic journals to politicized scientific messages transmitted in ordinary media (Atkinson, 1997; Dennen and Burner, 2007). In this variety of situations with different levels of complexity, the same rules of rigour apply, and students need to understand it. Broad epistemic integrity includes content that may be taught at different levels of complexity. It is already worthwhile to provide preliminary teaching at the stage of secondary school. Young students would benefit from learning about the importance of trustworthy information transmission and about how to evaluate the reliability of different sources of information. Nevertheless, we believe that university students would benefit the most because it will be of direct practical use to them. Indeed, at university level, students are expected to do high-quality research, notably while writing their undergraduate theses. They will be expected to find their own way through research literature. It is therefore a timely moment to teach them how to evaluate new data and navigate through complex arrays of information.

## 5 Efficacy of teaching broad epistemic integrity

In recent years, some philosophers and social scientists have expressed doubts about the efficacy of education in improving reasoning skills, critical thinking, or moral virtues (Deresiewicz, 2015; Caplan, 2018; Brennan and Magness, 2019). Although empirical research in these domains is scarce (El Soufi and See, 2019; Tuononen et al., 2022), we are not convinced that this scepticism reflects the overall trends reported in the scientific literature.

For instance, a recent meta-analysis on the impact of education concludes that one additional year of education has an average positive impact on intelligence of 1 to 5 IQ points (Ritchie and Tucker-Drob, 2018). This is an interesting indirect result since IQ is highly correlated with performance on the cognitive reflection test (CRT), which is associated with resistance to fake news (Bronstein et al., 2019; Pennycook and Rand, 2020).

Further, interesting results on the impact of ethics teaching tend to confirm that moral behavior can be improved with teaching. In two recent randomized controlled trials (an original study and a direct replication), Schwitzgebel and colleagues showed that classes on animal ethics led to a decrease of meat-eating at the local restaurant (Schwitzgebel et al., 2020, 2023). Due to the similarity of the pedagogical approach, there is reason to think that teaching open-mindedness or attachment to truth could also produce positive results.

## 6 Conclusion

In this paper, we have claimed that teaching broad epistemic integrity should constitute a fundamental aspect of any high school and university curriculum. Such teaching should target *epistemic skills* (mainly the training of metacognitive competencies and the application of helpful heuristics and basic methodological principles), and *epistemic virtues*, (including love of truth, intellectual humility, open-mindedness, and epistemic responsibility).

Our proposal should not be seen as an infallible solution to the problem of misinformation, but as an important element in a broader toolbox. In a recent article, Van Bavel and colleagues proposed four ways of fighting misinformation (Van Bavel et al., 2021): (1) fact-checking, (2) providing psychological resources, (3) removing bad actors, and (4) providing incentives for accuracy. This makes it clear that our focus on developing epistemic skills and virtues should not blind us to the necessity of complementary interventions (Bak-Coleman et al., 2022; Roozenbeek et al., 2022).

Broad epistemic integrity teaching will not produce perfect evaluators and producers of information. Students will still be sometimes (legitimately) lost in the flow of scientific information that they will encounter in daily life. But we should be thinking about students simply as one node in a network of epistemic agents. Improving the average accuracy of each citizen can have large effects, as this reverberates and ultimately leads to a large decrease in the transmission of inaccurate news. By teaching students how to be better evaluators of scientific information and faithful transmitters of that information, we are building a public that will in the long run improve through collective intelligence.

## Data availability statement

The original contributions presented in the study are included in the article/supplementary material, further inquiries can be directed to the corresponding author.

## Author contributions

AA: Writing – review & editing, Writing – original draft. CC: Writing – review & editing.
